# Integrated point-of-care testing (POCT) for HIV, syphilis, malaria and anaemia at antenatal facilities in western Kenya: a qualitative study exploring end-users’ perspectives of appropriateness, acceptability and feasibility

**DOI:** 10.1186/s12913-018-3844-9

**Published:** 2019-01-28

**Authors:** Nicole Young, Florence Achieng, Meghna Desai, Penelope Phillips-Howard, Jenny Hill, George Aol, Godfrey Bigogo, Kayla Laserson, Feiko Ter Kuile, Miriam Taegtmeyer

**Affiliations:** 1 Liverpool School of Tropical Medicine, Liverpool, UK; 2 Center for Global Health Research, Kenya Medical Research Institute, Kisumu, Kenya; 3 Division of Parasitic Diseases and Malaria and Center for Global Health, Centers for Disease Control and Prevention, Atlanta, GA USA; 4 Division of Global Health Protection, Center for Global Health, Centers for Disease Control and Prevention, Atlanta, GA USA

**Keywords:** Integrated health services, Antenatal testing, Point-of-care, HIV, Syphilis, Malaria, Anaemia, Appropriateness, Feasibility, Acceptability, Healthcare workers, Kenya

## Abstract

**Background:**

HIV, syphilis, malaria and anaemia are leading preventable causes of adverse pregnancy outcomes in sub-Saharan Africa yet testing coverage for conditions other than HIV is low. Availing point-of-care tests (POCTs) at rural antenatal health facilities (dispensaries) has the potential to improve access and timely treatment. Fundamental to the adoption of and adherence to new diagnostic approaches are healthcare workers’ and pregnant women’s (end-users) buy-in. A qualitative approach was used to capture end-users’ experiences of using POCTs for HIV, syphilis, malaria and anaemia to assess the appropriateness, acceptability and feasibility of integrated testing for ANC.

**Methods:**

Seven dispensaries were purposively selected to implement integrated point-of-care testing for eight months in western Kenya. Semi-structured interviews were conducted with 18 healthcare workers (14 nurses, one clinical officer, two HIV testing counsellors, and one laboratory technician) who were trained, had experience doing integrated point-of-care testing, and were still working at the facilities 8–12 months after the intervention began. The interviews explored acceptability and relevance of POCTs to ANC, challenges with testing, training and supervision, and healthcare workers’ perspectives of client experiences. Twelve focus group discussions with 118 pregnant women who had attended a first ANC visit at the study facilities during the intervention were conducted to explore their knowledge of HIV, syphilis, malaria, and anaemia, experience of ANC point-of-care testing services, treatments received, relationships with healthcare workers, and experience of talking to partners about HIV and syphilis results.

**Results:**

Healthcare workers reported that they enjoyed gaining new skills, were enthusiastic about using POCTs, and found them easy to use and appropriate to their practice. Initial concerns that performing additional testing would increase their workload in an already strained environment were resolved with experience and proficiency with the testing procedures. However, despite having the diagnostic tools, general health system challenges such as high client to healthcare worker volume ratio, stock-outs and poor working conditions challenged the delivery of adequate counselling and management of the four conditions. Pregnant women appreciated POCTs, but reported poor healthcare worker attitudes, drug stock-outs, and fear of HIV disclosure to their partners as shortcomings to their ANC experience in general.

**Conclusion:**

This study provides insights on the acceptability, appropriateness, and feasibility of integrating POCTs into ANC services among end-users. While the innovation was desired and perceived as beneficial, future scale-up efforts would need to address health system weaknesses if integrated testing and subsequent effective management of the four conditions are to be achieved.

**Electronic supplementary material:**

The online version of this article (10.1186/s12913-018-3844-9) contains supplementary material, which is available to authorized users.

## Background

HIV, syphilis, malaria, anaemia, and their co-occurrences are leading preventable causes of adverse pregnancy outcomes in sub-Saharan Africa (SSA) [1–8]. With 92% of the world’s HIV positive pregnant women, SSA has an estimated maternal HIV prevalence of 5.3% [9, 10]. Women with untreated HIV infection are eight times more likely to die during pregnancy or post-partum [1]. Without antiretroviral therapy, risk of mother-to-child transmission cumulates and reaches 25–45% over the gestation and breastfeeding periods, making early detection and viral suppression critical [11]. The African region has a maternal syphilis prevalence of 1.7%, translating to 63% of global syphilis infections in pregnancy [2]. Syphilis is associated with spontaneous miscarriage, stillbirth, preterm birth, low birthweight, neonatal death, and congenital infection in new-borns [12]. The duration of exposure in utero is a major determinant of foetal transmission and late treatment in pregnancy may not be effective against congenital disease [13]. Additionally, for sexually transmitted infections such as HIV and syphilis, involvement of male partners is necessary for effective care. In malaria endemic regions, 45% of pregnancies would have malaria infection without preventive measures [14]. Malaria is associated with anaemia, intrauterine growth restriction, preterm delivery, foetal loss, neonatal and infant mortality [15]. In Africa, prevalence of anaemia is high: among pregnant women in 2011, an estimated 46% were anaemic and 1.5% were severely anaemic [16]. Maternal anaemia is associated with fatigue, low-birth weight, and maternal and perinatal death [17]. There is evidence that risks of pre-term birth and low-birth weight are higher if maternal iron deficiency occurred before the second trimester of pregnancy than if it occurred later [18]. Moreover, these conditions are risk factors for one another and they often occur together [8, 19–22]. It is therefore essential to address these conditions together as early as possible during pregnancy, ideally with partner involvement, to protect mother and child [23].

The World Health Organisation has endorsed an integrated disease approach to antenatal care (ANC) that recommends testing for, preventing and treating these four conditions [24]. Despite this and the global success in increasing antenatal HIV testing coverage [25, 26], rates of testing for the other conditions are still low and few examples of integrated disease approaches to ANC exist at the programme level [27–29]. Kenya’s antenatal guidelines, which follow WHO’s recommendations, require testing for HIV, syphilis, febrile women for malaria, and anaemia at first ANC visits [30]. While over 95% of pregnant women attend ANC and over 90% of these women receive HIV testing [31], less than 50% are tested for syphilis or anaemia [32–35]. There are concerns that donor support to HIV programmes may have drawn attention away from non-HIV services [36, 37]. With the new Global Fund call for strengthening health systems through HIV funding, more synergies and integrated programmes can be achieved [37, 38]. Moreover, syphilis screening is one of the most cost-effective antenatal interventions, even in low prevalence settings [39, 40]. While there is currently no national recommendation to screen pregnant women for malaria infection unless a woman has a recent history of fever, screening with microscopy is commonly practiced in facilities with laboratories in malaria endemic regions in Kenya. Recently, neighbouring Tanzania has incorporated malaria testing at first visits into their antenatal policy [41]. There is also increasing interest internationally for a strategy that adds malaria testing at first visits to current recommendations of intermittent preventive therapy with sulfadoxine-pyrimethamine (IPTp-SP) and bed-net use [42–48].

Affordable and reliable point-of-care tests (POCTs) that require minimal training and no durable equipment are available for HIV, syphilis, malaria and anaemia and have been used successfully at the point-of-care [49, 50]. They have the potential to improve screening coverage at peripheral facilities and circumvent the need for referrals by shifting antenatal testing from laboratory technicians, who are often based at far centrally-located facilities, to nurses, midwives and lay healthcare workers at peripheral facilities. Referrals further delay testing when women already seek ANC late in pregnancy: approximately a third attend clinic in the first trimester, and a third in the third trimester [51]. POCTs have transformed antenatal HIV testing in SSA over the last two decades [52], alleviating barriers of stigma, relieving human resource shortages and reducing the need for repeated visits for test results or to reach laboratories [53]. Several studies that have assessed integrating syphilis POCTs with HIV testing for ANC have shown increased job satisfaction among healthcare workers, ease-of-use of tests and increased trust of diagnosis among pregnant women [54].

To examine the integration of syphilis, malaria, and anaemia point-of-care testing with antenatal HIV testing, we conducted an eight-month longitudinal implementation study from December 2014 to August 2015 among seven purposively selected rural facilities (dispensaries) within the study area of the Kenya Medical Research Institute (KEMRI) and Centers for Disease control (CDC) Health and Demographic Surveillance System (HDSS) in Siaya County, western Kenya [55]. These seven were selected based on geographical spread within the HDSS area, no other ongoing antenatal studies, antenatal care visit volume, and willingness to participate. Competency-based training was given to 23 healthcare workers: 14 nurses, two clinical officers, six HIV testing counsellors, and one laboratory technician. Training included how to run all four tests per standard operating procedures, safety and appropriate preventive and clinical management of positive results following Kenyan guidelines [30]. Quality assessments (QA) through observed proficiency testing of healthcare workers performing rapid tests compared to manufacturers’ instructions was done immediately after initial training and at three, six and nine months. Implementation outcomes of adoption and fidelity, using definitions from a conceptual framework by Proctor et al. [56], were measured quantitatively. In summary, we found that adoption was good as over 95% of pregnant women received all four POCTs at first ANC visit over the study period but treatment fidelity did not reach targets defined by the study. Healthcare workers’ minimum testing proficiency scores from quality assessments (QA) through performance observations improved from 70 to 91% over the study period, suggesting that most healthcare workers could accurately use integrated POCTs after remedial trainings and practice.

The intervention’s success in improving pregnancy outcomes rests upon healthcare workers’ willingness and ability to use four POCTs simultaneously during routine ANC visits, pregnant women’s early ANC attendance and their openness to testing and treatment, and partner involvement in the management of sexually transmitted infections. Further investigation was therefore needed to understand healthcare workers’ and pregnant women’s perceptions of the intervention’s appropriateness, acceptability and feasibility. Appropriateness and acceptability are the perceived relevance and agreeableness of the intervention respectively while feasibility is the extent to which the innovation can be carried out in the implementation environment [56]. Qualitative approaches were used to assess these implementation constructs: one-on-one semi-structured interviews (SSIs) with healthcare workers were conducted to capture individual experiences of providing integrated testing services and focus group discussions (FGDs) were done with pregnant women to stimulate sharing of experiences with ANC and blood testing services. Group discussions allow pregnant women to communicate naturally which can give insight into knowledge, attitudes and social norms as well as generate more critical comments perhaps because the group setting is less intimidating [57].

## Methods

### Data collection

We conducted healthcare worker interviews at the end of the implementation study, 8–12 months after the intervention began. All healthcare workers who were trained, had experience doing integrated point-of-care testing, and were working at the facilities during that time were interviewed (Table 1). A total of 18 healthcare workers (eight females, 10 males) were consented and interviewed in English by an experienced social scientist from the study area (FA) and a non-local researcher (NY) at the facilities. The lengths of time the healthcare workers have spent at their facilities ranged from one month to seven years. A semi-structured interview guide with open-ended questions was used to explore the intervention’s appropriateness, acceptability and feasibility (Additional file 1). The guide was designed to capture relevance of integrating POCTs to dispensary ANC services, challenges with testing, experience with training and supervision, experience with clients, and barriers to integration and health system constraints beyond healthcare workers’ control. Healthcare workers who have spent more time with the intervention were generally more verbose than those who were more recently transferred to the intervention facilities. The interviews lasted approximately 30–40 min.

**Table 1:** Baseline testing services and number of semi-structured interviews (SSIs) with healthcare workers and focus group discussions (FGDs) with pregnant women by facility

Facility	Turnover^a^	Volume^b^	Testing at baseline^c^				SSIs	FGDs
			HIV	Syphilis	Malaria	Hb^d^		
1	Low	High	Yes	No	Yes	Yes	1 F nurse, 1 M nurse	group 1: n = 5; group 2: n = 12
2	Low	Medium	Yes	No	Yes	No	1 F nurse, 1 M nurse	group 1: n = 9; group 2: n = 9
3	Low	Medium	Yes	No	No	No	1 F nurse, 1 M nurse, 1 M clinical officer	group 1: n = 10
4	Medium	Low	Yes	No	No	No	2 M nurses	group 1: n = 10
5	Medium	Low	Yes	No	Yes	No	1 F nurse, 1 M nurse, 1 M HTC	group 1: n = 9; group 2: n = 10
6	Low	Medium	Yes	No	No	No	1 F nurse, 1 M nurse	group 1: n = 12; group 2: n = 13
7	High	High	Yes	No	No	Yes	1 F nurse, 1 M nurse, 1 F HTC, 1 F lab tech	group 1: n = 11; group 2: n = 8

^a^Turnover of staff categorized into low, medium, and high defined as having 2, 1, and 0 skilled healthcare workers who received training at the start of the programme and remained for all 8 months of implementation respectively

^b^Facility volume was categorized as low, medium, and high for < 30, 30–40, and 50–70 monthly ANC visits respectively

^c^All 7 facilities’ routinely conducted HIV testing, 2 conducted anaemia testing irregularly, and 3 conducted malaria testing irregularly for ANC

^d^Haemoglobin test for anaemia

*M* male; *F* female; *HTC* HIV testing counsellor; *lab tech* laboratory technician; *Hb* haemoglobin; *ANC* antenatal care

We recruited ANC women at the intervention facilities during the first two weeks of August 2015, the last month of the implementation study, to allow healthcare workers enough experience with delivering point-of-care testing services. Women who had ever attended a first ANC visit at these facilities during the study period were invited (147 women) to participate in the FGDs. Twelve FGDs with 118 participating women were conducted at a location away from the facilities on a later date within the same month*.* One or two FGDs were held for each facility depending on the total number of women recruited (Table 1). The FGDs were conducted by local researchers to mitigate power and cultural differences between researcher and participants: FA led the discussions in the local language (Dholuo) and a trained moderator, also from the study area, took notes. Before the start of the discussions, consent forms were read together with the group and written informed consents were obtained from literate women and verbal consents with thumb prints and witness signatures were obtained from illiterate women. Women who participated in the FGDs ranged from 15 to 46 years old, one-nine gravida and from three weeks pregnant to recently delivered. A discussion guide in Dholuo was used to elicit pregnant women’s feelings and opinions of integrated point-of-care testing (POCT) services received (Additional file 2). The following domains were covered: knowledge of conditions, experience of ANC testing services and treatments received, relationships with healthcare workers, and experience of talking to partners. The topic guide was translated and back-translated from English to Dholuo and piloted before use to ensure consistent meaning across the languages. The level of interaction in the focus groups was generally well engaged and relaxed. However, there were quieter groups where the moderator had to stimulate more discussion among respondents using open-ended probes. Discussions lasted approximately one to two hours.

All interviews and FGDs were digitally recorded with consent. Local research assistants transcribed English interviews verbatim. They also transcribed non-English interviews in Dholuo and then translated them to English while FA made quality checks of the translations. All transcripts were anonymized. FA trained the moderator and research assistants in data collection and ethical considerations.

### Analysis

Regular meetings were held among the research team to review transcripts and notes from the interviews. Thematic analysis was used because we wanted to identify patterns of ideas across textual accounts of end-user experiences with POCTs in order to assess the intervention’s appropriateness, acceptability and feasibility. The process started with re-reading of transcripts to become familiar with the data. Segments of text that offered insight into user experiences were labeled by hand and the interpretations of these labels were discussed among two researchers (NY and MT) and a set of codes each for healthcare workers and pregnant women interviews were agreed upon. These coding frameworks were then applied to the rest of the transcripts. Any new codes were further discussed and incorporated into the framework. No new codes emerged after the first half of the data (nine SSIs, six FGDs) were analysed, indicating data saturation and further data collection was unnecessary. Coded data were collated and sub-themes around appropriateness, acceptability and feasibility of integrated POCT were formed. Viewpoints from healthcare workers and pregnant women were triangulated to create an overall narrative. The team then interrogated the findings against several existing conceptual frameworks describing the adoption of innovations into health systems [56, 58–61], and adapted them to best reflect the findings.

## Results

The findings were synthesized across the two participant types. Feasibility was inferred from participants’ views on barriers and enablers to delivering integrated testing and ANC services. Three sub-themes emerged from participants’ discussions: sub-themes related to community culture and concerns, local service delivery issues at the dispensaries, and wider health system organization (Table 2).

**Table 2:** Sub-themes of pregnant women and healthcare workers reflections on integrated point-of-care testing’s appropriateness, acceptability and feasibility

	Service delivery at dispensaries	Community context of cultures and concerns	Wider health system: policy, programme and management
Appropriateness	Healthcare workers’ professional motivations Services pregnant women want at ANC	Time and costs of reaching facilities	National guideline requirements
Acceptability	Healthcare workers: Complexity of POCTs Proficiency of testing Observability^a^ of test results Trialability^b^ of POCTs Workload Attitude towards gaining knowledge and new skills Pregnant women: Trust and confidence in results	Community stigma, gender violence and partner involvement	County level decision makers would need to value and prioritize integrated antenatal testing and allocate funds to ensure its continuity
Feasibility	Motivation of healthcare workers Drugs and commodities for services Training and quality assurance of healthcare worker performance Pregnant women’s degree of comfort in asking for services	Community culture and attitudes that influence timing of first ANC visit	Procurement and funding systems for commodities and drugs Sufficiency of human resources to meet demand Working conditions Quality and synergy of training

*POCTs* point-of-care tests

^a^Observability: the degree to which the results of an innovation are visible [61]

^b^Trialability: the degree to which an innovation can be experimented with on a limited basis so as personal meaning can be ascribed [61]

### Appropriateness of integrated POCT

#### Local service delivery and wider health system

##### Intervention enables healthcare workers to meet ANC guideline requirements but misses some essential tests:

All healthcare workers were excited about integrated POCT as it enabled them to meet pregnant women’s ANC needs in accordance with national guidelines.

*“At least we are able to manage ANC mothers wholly instead of us referring, you know we used to refer, even for Hb we used to refer to Siaya but at least now we can do all those.”*-female nurse (facility 1)

Some healthcare workers suggested enhancements to the programme such as retesting women’s haemoglobin (Hb) levels at revisits to monitor improvement if anaemia was detected at the first visit. Many were concerned that the intervention still missed urinalysis, blood grouping and rhesus tests and women still needed to be referred for these.

*“…but now when we were testing the mothers, there was this test Rhesus and urinalysis, how I wish they could add one for urinalysis so that you deal with the mother as a whole.”-*female nurse (facility 5)

Healthcare workers experienced the tangible benefits of testing, which allowed them to manage diseases like syphilis and malaria that they were not testing for before. They found anaemia testing to be of importance because they saw many women with low haemoglobin (Hb) readings.

*“Malaria, we were not also doing it but I think integration is better because … and these days you will get to find that, I usually get so many positives for malaria and Hb, I’ve even got Hb of 4.9. Now I was imagining if we did not have this Hb machine, like if you just test the mother for HIV as usual and tell her to go. Maybe she does not even have money to go to the District hospital to go for other tests, what will happen to that mother? I think that is what brings these maternal deaths because we do not take the precaution during the antenatal period.”* -female nurse (facility 6)

##### Intervention meets pregnant women’s intentions for going to ANC:

From the FGDs, pregnant women go for ANC to seek services which they believe are beneficial to the pregnancy. This includes finding out the position of the baby, learning how to take care of themselves during pregnancy, receiving help when there is a problem, and receiving medicines, diagnosis and treatment for harmful diseases. Women also mention choosing facilities based on proximity which makes availing point-of-care testing services at peripheral close-to-community facilities appropriate to pregnant women’s needs.

#### Community context

##### Addresses pregnant women’s need for time and cost savings:

Many healthcare workers cited the advantage of not having to refer pregnant women to laboratories for syphilis and anaemia testing as most do not go because of distance and transport costs, leaving vital conditions undiagnosed.

*“it was very challenging, you find the mother at the first visit, you tell them: go to diagnosis, go to district, go and test this and this. They don’t go. They don’t always go and the next day when they come it is just blank”* –clinical officer (facility 3)

Healthcare workers cited reasons such as poverty for why pregnant women do not go for referral services, which are usually farther away and would incur travel costs.

*“Sometimes there are some services which we do not offer which will force us to refer them to next level, of which because of the poverty in this area, some do not even go” *-male nurse (facility 1)

### Acceptability of integrated POCT

#### Local service delivery

##### Complexity of POCTs is acceptable and healthcare workers adapted to the intervention:

All healthcare workers found POCTs easy to use. Some healthcare workers felt like they had some initial difficulties, such as trouble obtaining enough blood for all four tests from a single finger prick (which was believed to be attributed to women having thick blood or low haemoglobin levels), remembering how to set the times on the triple timer, and controlling the pipettes. These issues were felt to be resolved with supervisory feedback and experience as they gained proficiency in the testing procedures.

*“I think the pipette for syphilis was challenging at first before I did some more training on it, using pipette for syphilis, that was the place the challenges were coming in, but we manage to fill the loop hole, I know how to use the pipette.” *-male HIV testing counsellor (facility 5)

The intervention’s trialability (degree to which healthcare workers were able to experiment with the intervention) allowed healthcare workers to ‘try-out’ the new testing procedures and adapt them to work under their own conditions which helped dispel initial uncertainty. Some healthcare workers were initially sceptical about the workload from doing extra tests, but these concerns resolved with experience, and developing individual strategies to integrate testing into workflows.

***“****When we were starting we also complained, we never wanted to be given more and there was no additional staff. We talked about that and finally we are able to cope. Yeah, we can just do them as we continue doing the other things.” -*male nurse (facility 4)

*“Yes, when it started I saw it was a lot of work, but now it has made it easy because I can perform all the tests on the table” -*female nurse (facility 5)

One healthcare worker re-organized her work to see first ANC women together for group counselling and group testing. She would perform the testing procedures for multiple women in an assembly line, starting the timer for the first woman and roughly estimating the extra waiting time for subsequent women. She requested that more timers should be given to accommodate testing multiple women together.

##### Integration of testing is more agreeable than separate testing points:

Some healthcare workers thought that conducting all the tests with one finger prick, as a one-stop-shop, improved client acceptability to testing and client flow.

***“****let me give an example to where I was, the difference to now, because there everything was done through the lab, but now here, they come here to VCT [voluntary testing and counselling] room, we do everything at once, so time management is adhered. And using one single prick, the client can get results at once. That is what the clients actually were complaining, if I’m testing for HIV and then they go again to be pricked for malaria, I think we were having a challenge, but since the IPOC came, we just do everything within one prick.”-*male HIV testing counsellor (facility 5)

These sentiments are reflected among pregnant women themselves who mention not liking to be pricked several times.

*“You can feel bad because when pricked many times and you have less blood you find that your blood level gets low*.”-pregnant woman (facility 5)

##### Healthcare workers were open to gaining knowledge and new skills:

Healthcare workers saw the programme as an opportunity to learn new skills and were eager to receive support such as training and supervision. Proficiency observations and remedial training were especially appreciated as they served as corrective reminders for forgotten procedures, which supported POCTs’ continued use. However, one healthcare worker qualified that the attitude of the supervisors should be positive and constructive rather than fault-finding. Many healthcare workers were happy to learn how to use syphilis POCTs and the HemoCue machine, which they had not used before. One clinical officer requested more support as he felt on-site training was less sufficient than multi-day central training and found it difficult to perform with the same quality as those who attended multi-day trainings. Because more conditions were tested for, some healthcare workers felt they needed more training on counselling.

##### Observability of tests results improves trust in diagnosis:

Healthcare workers liked the ease of observing test results as it helped them communicate the diagnoses directly.

*“but they will not take it very serious because they have seen you have not even tested but when I test them, I always show them and tell them your Hb is this and you are supposed to have this. At least the mother can now understand because she has read it directly from [the machine] and I have explained they should be at what level, in fact they take it very serious.”* -male nurse (facility 2)

Pregnant women were taught to observe their own results from the test cassettes which increased their trust of diagnoses.

“*I always know because if you go there [health facility], and maybe they are doing an HIV test, if it comes out one line after testing it’s negative and if they are two then you are positive. So I know very well.”-* pregnant woman (facility 6)

“*You cannot deny the results because after the tests, they are only yours; they are not combined with another person’s results to make you doubt that it is someone else’s. So when the results are back, you have to check to see if it is negative or positive. If it is found to be negative, you just take it to be yours, you will not deny.”* -pregnant woman (facility 7)

However, a few healthcare workers mentioned problems with the HIV tests, which sometimes gave faint positive lines, making women question the validity and deny the results.

*“we had some problem with it [HIV test] and actually…in interpreting the results, it used to give some faint line at the test site. It was causing a lot of problems with the clients. They are saying the line was faint.”* -male nurse (facility 2)

#### Community context

##### Stigma and difficulties of partner involvement:

Some women mentioned there is still stigma and fear around HIV testing while syphilis, malaria and anaemia testing were not mentioned to be threatening.

“*Some women are afraid that they will be tested for HIV and they are afraid, so they prefer going to the CHW [community health worker]*.”- pregnant woman (facility 5)

Women suggested that information and counselling would help with acceptance of HIV diagnostic test results and treatment compliance.

*“They do counselling well for you to accept the result just as she had mentioned earlier that you see the test results yourself and confirm that they are yours and if added with counselling you just feel comfortable.”-*pregnant woman (facility 5)

Many women emphasize the importance that adequate counselling be given to assuage fears regarding positive test results for HIV.

*“Sometimes you are given enough counselling, sometimes not. When you are well counselled you feel okay but when you are not told enough you feel worried, so you can also ask to know. So you should just be free to ask them so that you go back feeling okay.*”-pregnant woman (facility 7)

*“When you are found to be having the illnesses, whoever was with you should sit with you and counsel you so that you can be at peace*.”- pregnant woman (facility 3)

While all women believed that partners should be tested and treated for HIV, they stated that most of their male partners could not be convinced to be tested because of fear. Some women recounted tricking their husbands into going for HIV testing.

“*I have told my husband to take me to the clinic as a motorcyclist so when we arrived I told him it is just for a few minutes he should wait for me and after the doctor was done with me I lied to him that the doctor was calling him so that he can be told something and when we reached at the doctors place I told him that I want us to be tested to know our HIV status so there was no way he could leave me here alone (laughing). We were tested both of us now*.”- pregnant woman (facility 5)

Other women said some partners would assume they shared the same test result as their wives and some even share the women’s HIV drugs.

“*If you are found to be positive, it is important to test the spouse also so that they also know their health status. Most of them do not accept. So they rely on your results saying that if you are positive, they are also and if you are not, they also are not.*”- pregnant woman (facility 7)

“*They check your ANC book, so if you are negative they also know that they are negative*.”- pregnant woman (facility 3)

Women also reported that they feared blame and violence from their husband if they disclosed their HIV positive status and their low social status makes it harder for them to protect themselves.

“*You can find some men, after knowing your status and the doctor has given you protective measures, some men do not accept to use the condoms. They say they cannot use them and if you also deny, some really get violent and beat you up*.”- pregnant woman (facility 7)

“*You just keep silent because you stay in his house, in their home, in their land*”- pregnant woman (facility 7)

### Feasibility of integrated POCT

#### Local service delivery and wider health system

Healthcare workers and pregnant women talked about their experiences at facilities which affect the delivery of ANC services, including diagnostic testing, with implications that concern the health system organization.

##### Working conditions compromise the quality of ANC given to pregnant women:

Although healthcare workers felt the testing procedures were acceptable, there were pervasive sentiments of frustration and impatience from having extra clients to see when other healthcare workers were absent. This was felt to compromise on quality of care, such as worse healthcare worker attitudes, and not giving adequate counselling.

*“if you are stationed in MCH that is ok, but if you are taking care of PSC [patient support care for HIV positives], taking care of outpatient clients…you don’t give quality, let me say this.”* -male nurse (facility 2)

*“Sometimes you are here alone, you are doing all the work in the facility, so you work until you are tired because first of all you have the patients themselves and sometimes they can be very many they reach even 100 of them and you are alone. You are the one who is doing the malaria testing, you are the one who is prescribing and you are the one issuing the drugs, you also have the antenatal mothers and sometimes you can also have a mother in labour- those are some of the challenges we get so sometimes I’m sure we don’t even give quality work (laughing). So when things are that way you just think of chasing everybody out of the compound if they have nothing to do. You do things at a very fast speed because now you even don’t want time with the patient and you have talked such that you don’t feel like asking the patient her problems because you are tired, that is something I have experienced and then also if you have that burn out the patients will say that the sister is talking badly but I normally tell them it’s because I’m tired, that is the problem*.” -female nurse (facility 2)

Some healthcare workers felt the 20-min waiting time for test results was too long when there were many clients waiting.

*“The timer now, you know consume a lot of time, you have to wait for like 20 minutes and you have a long queue so there is a bit of a challenge there*.” -male nurse (facility 7)

This is echoed by most pregnant women who recounted they received very little counselling which was attributed to shortage of staff.

“*Because there are few doctors here, we do not get enough counselling because there are many patients who are being attended to by one person. When the doctors were two, they would give enough counselling when they are free*.” -pregnant woman (facility 4)

Moreover, poor healthcare worker attitudes negatively affect pregnant women’s ANC experiences.

“*Pregnant women can avoid coming to the clinic because they are afraid of meeting the healthcare workers*”- pregnant women (facility 1)

“*But you know when we come to the clinic we find different types of nurses sometimes you find her not in the moods and you get afraid of asking but if you find a happy one then you are also happy such that you share with her your problems.” -*pregnant woman (facility 6)

##### Stock-outs of commodities and drugs made it difficult to provide care:

Frequent shortages of drugs made healthcare workers unable to provide treatment after diagnosis which made them feel frustrated because they could not treat the client.

*“…how can you work without supplies that means you will go to the health facility and sit because clients need to be helped because they need help and they come, you diagnose them and you don’t help them with the drugs.” -*male nurse (facility 6)

*“You feel so bad when a mother comes and you ask her to buy it [drugs]. When she comes for the next visit, you ask her, did you buy? She will tell you no, I did not buy. I went to the chemist and they told me they sell it at 150 shillings and I did not have the money. Now you feel so bad, that is why I decided to buy the first batch but I can’t continue buying. Because we are not paid and we are paid, in fact poorly. So I cannot also manage to buy it, it is as if I am becoming another NGO.” -*female nurse (facility 6)

Gloves were also in shortage, making it difficult for healthcare workers to protect themselves when doing testing.

*“Right now we are having shortage of gloves, so it is forcing us to work under, let’s say under pressure, because if there is no gloves sometimes it is hard to work in such conditions” -*male HIV testing counsellor (facility 5)

Healthcare workers were frustrated about the devolution of government control and management to the counties and thought it to be the cause of stock-outs:

*“it has been worse…things have changed…when we were still under the national government, drugs used to come to facilities…and they were punctual…but nowadays, you can even stay for months without drugs and when they bring in drugs, they bring that which you are going to use for about two weeks.”* -female nurse (facility 6)

*“Actually… personally I believe that health should have been devolved much, much later but it was rushed, and you can see, the management is poor. Just the other day, health workers within the county were threatening to go on strike. And part of the problem is just the management. I think some of these staff was devolved from the national government when the county government had not put in the structures to manage this work force from the national government and probably make some input. And again, also the input of the county government in terms of even staff employment, supplying of drugs … all these like I told you earlier that we have experienced shortage of drugs for quite a long time.”* -male clinical officer (facility 3)

Drug shortages left pregnant women untreated because they could not afford to go to pharmacies to purchase drugs for themselves:

*“There is no right treatment given to us because you can be prescribed for drugs and you don’t have money to go and buy them so there is no good treatment given.” *-pregnant women (facility 2)

##### Inadequate training and supervision:

There were some healthcare workers who mentioned never having received training for HIV and malaria testing even though these are among the commonly used tests at dispensaries. One nurse said she was grateful for POCT training because before she would directly squeeze blood from the finger to the cassette for HIV testing as she did not know how to use the pipette. Another reported she had never used, or seen people use, timers for malaria POCTs and the training had enlightened her on the importance of timing so that she can now trust the results.

*“It is good because at least you follow the right procedures. It also helps in getting the correct result because in IPOC I know there is timing of the results and the [not clear] measures. Because before IPOC came, I didn’t know anything to do with timing of the… I knew there was timing but the timer was not there. Yeah, so the results maybe, you give the result before time of which the results can be inaccurate. Yeah, and also the procedures at least they follow up on how to do the procedures so that we can do them correctly.” -*female nurse (facility 7)

A small minority of respondents described that currently in western Kenya, HIV and PMTCT services are supported by international partner programmes and receive frequent on-site mentorship, while other maternity services, supervised by the Ministry of Health, rarely receive this level of support.

#### Community context

##### Delayed or avoidance of ANC attendance:

Pregnant women’s first ANC visit was often late in the pregnancy and the potential effectiveness of integrated POCT was thought by healthcare workers to be undermined by the delayed management of conditions.

Women explained that late attendance was because they did not like to go to ANC before their pregnancy was certain, which was around the fourth month. Additionally, they stated that long distances, the cost of transport, poor staff attitudes, and need for multiple revisits were reasons women avoided ANC.

***“****Some women are afraid of walking while pregnant and since the CHW [community health worker] is next to her than the hospital she prefers going to the CHW because she can always go there once in seven months but in hospital they will tell her to go to the hospital every month and that doesn’t make her happy so she goes to the CHW.”* -pregnant women (facility 5)

***“****We should start attending the clinic at four months and it should be every month without fail so that we can be helped. At four months you will have known your status in pregnancy.” -*pregnant woman (facility 6)

“*Some also don’t go to the hospital because they think they will be asked money*” -pregnant woman (facility 5)

## Discussion

This study presents healthcare workers’ and pregnant women’s experiences on using POCTs to diagnose HIV, syphilis, malaria and anaemia at dispensaries in western Kenya. The intervention was perceived to be highly acceptable and appropriate: healthcare workers felt positive about offering POCTs and pregnant women appreciated the extra services. Healthcare workers were enthusiastic about learning new skills and to be able to provide more holistic services to pregnant women, suggesting that availing services may improve motivation. Data from elsewhere suggest that innovations are more likely to be adopted and implemented if they are easy to use, appropriate to the practice setting and accompanied by training and feedback support [56, 59, 61]. Overall, healthcare workers found POCTs user-friendly, aside from a few technical challenges that were addressed through experience and remedial training, which led to greater confidence in using the tests. This experience highlights the importance of supervision and audit with feedback, even for uncomplicated innovations, for improving performance [62, 63]. Findings from this study are also reflected in other studies on implementation of POCTs where they have shown that healthcare workers and pregnant women appreciate their simplicity, which reduces diagnosis time [64–66]. A common finding from these studies is that the greatest bottleneck to scale-up is not in the satisfaction with the tests, but rather weakness of the wider health system. Test stock-outs, inadequate training and supervision, human resource constraints, and vertical funding structures have undermined scale-up of rapid syphilis testing in ANC [27, 67]. Similar challenges with implementing malaria POCTs have also been reported [68–70]. Sustaining a skilled, motivated and well-supported workforce with adequate commodities to effectively deliver integrated services, beyond the end of the study intervention period, will require effort on several levels. An integrated supervision and monitoring solution to support holistic ANC would also be needed.

For the intervention to be feasible, adequate health system support is needed to ensure resources, such as trained healthcare workers and commodities, are available. Despite the prominent health burden of syphilis, malaria and anaemia and the availability of simple cost-effective solutions, interventions to address these illnesses at ANC have been poorly delivered in Kenya and elsewhere in SSA, compared to HIV programmes [27, 28, 35, 40, 71, 72]. International advocates play a significant role in norm promotion and shaping countries’ policy preferences [73, 74]. Syphilis and haemoglobin screening, despite the strong evidence for their clinical effectiveness [40, 50, 75], lack donor advocacy and consequently achieve less coverage [27, 67, 76]. More transnational backing to shift the paradigm from single-focused programmes to comprehensive maternal health care is needed [77].

During the time of this study, the Kenyan government was politically motivated to rapidly execute the decentralization process of transferring decision-making power from the central government to 47 newly-formed counties after 2013 elections [78]. In the devolved system, health service delivery functions are placed under the jurisdiction of the county governments [79]. Unfortunately, poor organization and rapid execution of the decentralization process resulted in widespread confusion and unpreparedness for the structural changes in finance and administration [78]. The county governments had limited technical capacity to set priorities and allocate funds, which created challenges in the county’s financial flows during the study (three years since devolution), causing stock-outs and funding delays [78]. For an integrated testing strategy to be sustained beyond the end of the study, county authorities involved with selecting priorities will need to understand the importance of antenatal screening and be willing to allocate funds towards it. Healthcare decisions since devolution increasingly involves local politicians and technical actors; it is important to engage with them, as well as women within the community, to highlight the equity benefits associated with an integrated testing strategy at peripheral facilities that can extend diagnostic services to a much larger demographic of women.

Healthcare workers suffer low morale from meagre and delayed salaries, lack of choice in placement, job grade stagnation, and feeling helpless due to stock-outs of commodities and drugs [80–82], resulting in recurring healthcare worker strikes to demand for better wages and working conditions [83]. Poor healthcare worker management, and the resulting frustrations can lead to low productivity, poor quality of care and cultures of predatory provider-client relationships [62, 84, 85]. Low morale among healthcare workers and resulting poor attitude have previously been found to lead to attrition of pregnant women from the HIV testing and treatment cascade, non-disclosure of status during delivery, and knowingly forfeiting nevirapine for new-borns [86]. All healthcare workers interviewed expressed concerns about workload. This is a common problem in SSA health systems in where critical workforce shortages, skill mix imbalance and mal-distribution have been described as a serious obstacle to scaling up priority interventions [80]. Additionally, disease-specific short training, polio eradication campaigns, and staff leave often pull already strained personnel away from facilities [77, 80, 87], leaving staff ‘alone’ to manage all the clients.

This integrated POCT strategy addressed a facility-level accessibility coverage gap by bringing essential testing services to peripheral facilities, circumventing the need for women to be referred to distant facilities for testing. However, it still rests upon the condition that women attend ANC at these facilities in the first place, attend early enough, and involve partners in the treatment of HIV and syphilis. Late attendance to ANC precludes preventive measures and effective treatment to protect the foetus. Our research has revealed that poor relationships between pregnant women and health providers, pregnancy uncertainty and the number of revisits required for the full ANC schedule of visits were reasons given by pregnant women for delaying ANC initiation. Other factors such as age, parity, money for transport, ignorance of gestation age to start ANC, not having any perceived problems with the pregnancy, and social pressures have previously been reported as reasons for delaying the start of ANC [88–91]. Distance was cited as the main reason for not attending ANC in last pregnancy in the 2012 Kenya AIDS Indicator Survey (KAIS) [31]. Addressing these determinants from the community through a community health strategy may improve ANC usage [92, 93].

HIV and syphilis management require involvement from male partners and ANC has not been good at capturing male populations [94]. Male involvement has been shown to improve women’s acceptance of HIV care and treatment [95], which is necessary to prevent re-infection of sexually transmitted infections. Our findings suggest stigma, fear of spouse violence, and cultural beliefs that men do not participate in reproductive health programmes impede male partner involvement. Interviews with men have suggested that men avoid ANC because of healthcare worker’s harsh attitudes [96]. Strategies to address gender dynamics and create male friendly experiences of women’s reproductive health are needed.

There were several limitations in this study. Our interviews and discussions were limited to frontline users and did not include management and implementation partners at county and national levels who have ultimate policy setting and budgeting decisions. Stakeholder insights, priorities and engagement are necessary to assess sustainability, readiness and intention for scale-up. Their insights will form a critical area for future research. We also did not rank the interviewees’ perceived significance of each barrier with participatory methods, which would have been useful to help future programmes prioritize resources. The SSIs and FGDs were also conducted towards the end of the implementation study and may not have captured early adoption experiences. Self-reported information is inclined to have desirability or courtesy bias, which we tried to minimise by emphasizing anonymity and conducting the focus groups away from the facility. Healthcare worker interviews elicited several critical comments which suggests that the courtesy bias is not limiting. Finally, our study was small, only involving seven facilities in western Kenya, which may not have captured the diverse health system environments in the SSA region.

## Conclusions

The study provides insight on the acceptability, appropriateness, and feasibility as well as potential explanations of high adoption and low fidelity of the integrated testing programme among end-users. Healthcare workers and pregnant women found point-of-care testing to be an important and necessary intervention for antenatal care. Recommendations for scale-up efforts include the need to address vertical programming structures by engaging donors and programme managers to encourage horizontal thinking of disease management and to create synergies across programmes. Human resource challenges will need to be addressed to improve healthcare worker conditions and commodity supply chains strengthened to ensure no stock-outs. Community engagement to encourage early ANC attendance is needed for early detection and treatment of conditions. Strategies to involve male partners and decrease stigma of HIV would help create openness and acceptability to care and treatment as well as safeguard pregnant women against gender violence. This will require a holistic effort from the community, county, national and international leaders.

## Additional file

Additional file 1: Semi-structured interview guide for healthcare workers (DOCX 17 kb)

Additional file 2: Focus group discussion guide for pregnant women (DOCX 22 kb)
