# Supplementary material for: Integrated point-of-care testing (POCT) for HIV, syphilis, malaria and anaemia at antenatal facilities in western Kenya: a qualitative study exploring end-users’ perspectives of appropriateness, acceptability and feasibility
Source: BMC Health Serv Res. 2019 Jan 28;19:74. doi: 10.1186/s12913-018-3844-9 (PMC6348645; doi:10.1186/s12913-018-3844-9)
Supplement: Supplementary file 1 — Semi-structured interview guide for healthcare workers (DOCX 17 kb) [file 12913_2018_3844_MOESM1_ESM.docx]

**HEALTHCARE WORKER INTERVIEW GUIDE**

**Appropriateness, acceptability and feasibility of integrated point-of-care testing for HIV, syphilis, malaria and anaemia in level 2 facilities in western Kenya**

**Interview Guide**

**Objectives:** To explore health workers’ perceptions and experiences of implementing integrated point of care testing services for HIV, syphilis, malaria and anaemia

A. Introduce the purpose of the study – its aims and scope

 Assure participant of confidentiality and how it will be maintained

 Ask for their consent to participate (fill in consent form)

B. Note details of participant.

| 1. Interviewee ID |  | 6. Gender | Male  Female  |
| --- | --- | --- | --- |
| 2. Date of Interview |  | 7. Age |  |
| 3. Name of interviewee |  | 8. # of children |  |
| 4. Province interviewee is from |  | 9. Education level |  |
| 5. Marital status |  |  |  |

***General questions***

1. Tell me a bit about yourself? How did you come to work in the health field?
2. How long have you been working at this facility?
3. What do you like and dislike about it?
4. How long do you think you will continue to work at this facility?
5. Are healthcare workers transferred often? What determines how they are transferred?
6. Has things changed since the devolution of management to the county government? How?
7. People are striking now, what are the main reasons for the strike?
8. What are the main challenges you face in your professional life?
9. How do you cope with them?

***Point-of-care testing***

1. How do you find doing IPOC testing for pregnant women?
2. Did your perspective change from before you started until now after 8 months? How?
3. Before IPOCT what kind of testing was done at this facility?
4. Who does it?
5. Did you receive any training from it?
6. Are there regular QAs? How often is the QA done and by who?
7. What are some of the challenges you face in offering IPOC to ANC mothers?
8. Which part do you find most challenging on a scale of 1-5; 1 being easiest and 5 being most difficult?

Getting blood for all 4 tests; Using the timer; Using the pipettes; Waiting time to read negative results; Observing safety with gloves, sharps container, biohazard bin liners; Telling pregnant women the results; Counselling

1. Currently ANC mothers are tested at their first ANC visit. What do you think about retesting them at subsequent visits?
2. How were you trained for IPOC?
3. How did you find the training?
4. Are there any skills you would have liked more training on?
5. If a new health worker reported, how do you feel about training him/her in IPOC?
6. How do you find the quarterly QA?
7. How do you find the integration placemat?
8. How can it be improved?
9. Many women do not know what their blood level means. Do you have time to tell them about their Hb level?
10. What do you do for a woman who has low Hb?
11. Many women do not know what syphilis is around this area?
12. Do you have time to tell them about syphilis?
13. What do you do when a woman is found to be syphilis positive?
14. Sometimes when a woman is tested positive for HIV, she doesn’t accept her status immediately. How do you handle such a case?

**Ways to probe:**

• Would you give me an example?

• Can you elaborate on that idea?

• Would you explain that further?

• I’m not sure I understand what you’re saying.

• Is there anything else?
• What do you mean by that?
