# Supplementary material for: Integrated point-of-care testing (POCT) for HIV, syphilis, malaria and anaemia at antenatal facilities in western Kenya: a qualitative study exploring end-users’ perspectives of appropriateness, acceptability and feasibility
Source: BMC Health Serv Res. 2019 Jan 28;19:74. doi: 10.1186/s12913-018-3844-9 (PMC6348645; doi:10.1186/s12913-018-3844-9)
Supplement: Supplementary file 2 — Focus group discussion guide for pregnant women (DOCX 22 kb) [file 12913_2018_3844_MOESM2_ESM.docx]

## Focus group discussion guide for pregnant women who completed first ANC visits in participating antenatal clinics (ENG)

FGD ID No [___]___]___] Moderator Initials [___]___]___] Note-taker Initials [___]___]___]

Recorder Number: _____________ Folder/File Name (location on recorder): _________________

Date [___]___]/[___]___]/[___]___] Interview location _________________________________

Time: Start ____________________ Time: Stop________________________ Duration:_________

No. Participants at start of FGD: ___________ No. Participants at the end of FGD: _____________

Introduction

I am ___________________________________from ________________________(moderator)

I am ___________________________________from________________________(note-taker)

**General purpose of the study**

• We are trying to find out what health checks and blood tests are done in the antenatal clinic, what you understand and are told about these checks in the clinics and what care you get if the tests show you need treatment. We would like to know about the care you and other pregnant women have experienced and in particular what difficulties you experience when you try to get the care you need. This information will help us understand what issues are important to you and other pregnant women, and if there are any problems.

**Aims of the interview and expected duration**

• Perceptions of ANC service and health workers

• Perceptions of HIV, syphilis, malaria and anaemia in pregnancy

• Perceptions of blood tests and treatment

• Identify best way to provide diagnosis and treatment at a single ANC visit.

• We will have a discussion that will from 1-2 hours

**Who is involved in the process (other participants)**

• We will hold discussions with women early in their pregnancies who have visited one of the

antenatal clinics for their 1st ANC visit.

**Why the participant’s cooperation is important**

• We are asking you to help us better understand what health checks and blood tests are now

done in pregnancy, and if pregnant women receive the treatment that they need. We want to learn about what more you think is needed for antenatal care and what you do when you get ill. We will also talk about care that is needed for pregnant women with health needs such as infections with HIV and syphilis. We also want to know about the experiences of you and other pregnant women with local health staff or other providers of health care or medicines, such as traditional healers or shop keepers. This knowledge will help us understand how to improve the care available in your community. What will happen with the collected information and how the participant/target group will benefit

**Ground rules:**

• Only one person talks at a time.

• It is important for us to hear everyone’s ideas and opinions. There are no right or wrong answers

to questions – just ideas, experiences and opinions, which are all valuable.

• You do not need to share experiences that make you feel uncomfortable. You can leave at any

time.

• It is important for us to hear all sides of an issue – the positive and the negative.

• Confidentiality is assured. “What is shared in the room stays in the room.”

• Turn off mobile phones

**Any questions?**

**Consent to record**

We would like to use a tape recorder so that information is collected correctly – please let us know if this is acceptable?

Please note the questions here:

___________________________________________________________________________________

___________________________________________________________________________________

___________________________________________________________________________________

___________________________________________________________________________________

**READ CONSENT FOR AND OBTAINED INFORMED CONSENT**

Demographic information for every FGD participant [to be completed on a one-to-one basis, immediately after consent is obtained]

Participant no. Age Date of 1st ANC Gravidity Gestation age Marital status Education Ethnic group

___________________________________________________________________________________

___________________________________________________________________________________

___________________________________________________________________________________

___________________________________________________________________________________

___________________________________________________________________________________

___________________________________________________________________________________

___________________________________________________________________________________

___________________________________________________________________________________

___________________________________________________________________________________

___________________________________________________________________________________

___________________________________________________________________________________

___________________________________________________________________________________

MODERATOR: Allow group to decide; assign fake names or use participant numbers. Make sure to use these fake names or participant numbers in note-taking and transcription. Make name tags using the fake names or participant numbers.

* REMEMBER – you MUST ask same questions for all groups or you are biasing results

COMMENTS – reasons for withdrawal, refusal, ambience of FG, level of interest, disagreements, etc

Warm up

Can we go around the room and say which village you are from and whether this is this your first pregnancy?

Now I am going to introduce some topics I hope you can discuss together, about your experiences during pregnancy.

| **Opening questions and Probes** |
| --- |
| **SERVICES RECEIVED AT ANC**   1. **You all recently attended ANC at ____. Why do pregnant women choose to attend ANC at _____?** 2. **Are you aware of what should be given to pregnant women at their first ANC visit? Can you tell us? Did you receive these services?**      1. **Do pregnant women feel comfortable in asking for services they know they should have but did not receive from the healthcare worker?** 2. **Do you know the blood tests that should be done for pregnant women at their first ANC visit? Can you mention them? What kind of blood tests were pregnant women given at the ANC at _____?**      1. **What was your experience about counselling offered at the ANC?**   Probe: HIV counselling, blood test counselling, birth plan counselling, nutrition counselling |
| **KNOWLEDGE OF HIV, SYPHILIS, MALARIA AND ANAEMIA AND THE BLOOD TESTS**   1. **What do you know about HIV and how it affects the pregnant women and the unborn child?**   Probe: risk of infection during pregnancy, risk of infection during childbirth, risk of infection during breast feeding?   1. **What do you know about syphilis and how it affects the pregnant women and the unborn child?**   Probe: Assess knowledge of syphilis (A bacterial infection usually spread by sexual contact/blood transfusion/congenital, that starts as a painless sore on genitals, mouth or rectum. If transmitted to the baby during pregnancy, can cause malformations)   1. **What do you know about malaria and how can it affect the pregnant women or the unborn child?** 2. **What do you know about anaemia and how it can affect the pregnant women or the unborn child?**   Probe: knowledge of different levels of anaemia   1. **What was pregnant women’s experience of the blood tests they do at the ANC?**   Probe: How do you feel about the amount of explanation received about blood tests given by  healthcare worker? How many times were the fingers pricked? Is that acceptable?   1. **How do pregnant women feel about the amount of explanation received about a positive result from the blood tests if done?**   Probe: How do the healthcare workers communicate the results? Do women understand what  the results mean for them and their baby? Do pregnant women accept the results?   1. **If a blood test result is positive, what should be done for pregnant women?** 2. **For HIV? Are pregnant women given the treatment they need?**   Probe: Do healthcare workers give enough counselling to pregnant women so they can  accept their results? When are pregnant women given care for HIV?   1. **Syphilis? Are pregnant women given the treatment they need?**   Probe: Do pregnant women know the right treatment for syphilis? Do healthcare workers  give adequate explanation for the treatment?   1. **Malaria? Are pregnant women given the treatment they need?**   Probe: Availability of quinine, co-artem in facilities? Availability of SP? Given bed-nets?  Advised to sleep under bed-net?   1. **Anaemia? Are pregnant women given the treatment they need?**   Probe: Do the healthcare workers tell pregnant women their blood level and give  advice/treatment? Availability of iron/folic acid? Do pregnant women know the different  levels of Hb and how much iron pills to take depending on blood level? Do pregnant women  experience side effects of the iron pill? Are pregnant women referred for blood transfusion if  blood level is very low?   1. **If positive for HIV or syphilis, it is important to treat the spouse as well. What is pregnant women’s experience of talking to their spouse about testing and treatment at the health facility?** |
| **QUALITY OF SERVICES RECEIVED**   1. **What are pregnant women’s experiences of the ANC at _____?**   Probe: How did you feel about the waiting time? Did you receive everything you expected? Do they prefer male or female health workers?   1. **How do the healthcare workers at ____ treat pregnant women?**   Probe: What advice do they give pregnant women? How is the attitude of the healthcare workers? Do you spend enough time with the healthcare workers? How do healthcare worker attitude influence where pregnant women seek advice?   1. **Were pregnant women asked to pay anything?** 2. **Do pregnant women feel they can discuss their pregnancy with the healthcare worker?** |
| **Closing**  We are now approaching the end of our discussion. Is there anything else anyone would like to add about the blood tests that you get from ANC or health facilities that we have not talked about?   - Summarise - Thank participants   Provide extra information and contacts to participants |
